# Serum Polyunsaturated Fatty Acids Correlate with Serum Cytokines and Clinical Disease Activity in Crohn’s Disease

**DOI:** 10.1038/s41598-019-39232-z

**Published:** 2019-02-27

**Authors:** Elizabeth A. Scoville, Margaret M. Allaman, Dawn W. Adams, Amy K. Motley, Shannon C. Peyton, Sarah L. Ferguson, Sara N. Horst, Christopher S. Williams, Dawn B. Beaulieu, David A. Schwartz, Keith T. Wilson, Lori A. Coburn

**Affiliations:** 1 Division of Gastroenterology, Hepatology, and Nutrition, Department of Medicine, Vanderbilt University Medical Center, Nashville, TN USA; 2 Department of Pathology, Microbiology, and Immunology, Vanderbilt University Medical Center, Nashville, TN USA; 3 Vanderbilt Center for Mucosal Inflammation and Cancer, Department of Medicine, Vanderbilt University Medical Center, Nashville, TN USA; 4 Veterans Affairs Tennessee Valley Healthcare System, Nashville, TN USA; 5 Vanderbilt Center for Stem Cell Biology, Vanderbilt University Medical Center, Nashville, TN USA; 6 Vanderbilt Ingram Cancer Center, Vanderbilt University Medical Center, Nashville, TN USA

## Abstract

Crohn’s disease (CD) has been associated with an increased consumption of n-6 polyunsaturated fatty acid (PUFA), while greater intake of n-3 PUFA has been associated with a reduced risk. We sought to investigate serum fatty acid composition in CD, and associations of fatty acids with disease activity, cytokines, and adipokines. Serum was prospectively collected from 116 CD subjects and 27 non-IBD controls. Clinical disease activity was assessed by the Harvey Bradshaw Index (HBI). Serum fatty acids were measured by gas chromatography. Serum cytokines and adipokines were measured by Luminex assay. Dietary histories were obtained from a subset of patients. Nine serum cytokines and adipokines were increased in CD versus controls. CD subjects had increased percentage serum monounsaturated fatty acids (MUFA), dihomo-gamma linolenic acid (DGLA), eicosapentaenoic acid (EPA), docosapentaenoic acid (DPA), and oleic acid, but decreased arachidonic acid (AA) versus controls. The % total n-3 fatty acids and % EPA directly correlated with pro-inflammatory cytokine levels and HBI, whereas the % total n-6 fatty acids were inversely correlated with pro-inflammatory cytokine levels and HBI. CD subjects had increased caloric intake versus controls, but no alterations in total fat or PUFA intake. We found differences in serum fatty acids, most notably PUFA, in CD that correlated both with clinical disease activity and inflammatory cytokines. Our findings indicate that altered fatty acid metabolism or utilization is present in CD and is related to disease activity.

## Introduction

Crohn’s disease (CD), a type of inflammatory bowel disease (IBD), is characterized by relapsing, remitting chronic inflammation that can affect the entire gastrointestinal tract. The pathogenesis of CD is thought to involve multiple predisposing genetic, environmental, and immunologic factors^1–4^. Epidemiologic studies have related increased animal fat and n-6 polyunsaturated fatty acid (PUFA) intake with the prevalence of both CD^5,6^ and ulcerative colitis (UC)^7^. In addition, increased intake of n-3 PUFA has been associated with a reduced risk of CD^8^. Genetic polymorphisms associated with alterations in the metabolism of long chain PUFA from dietary linoleic acid (LA) and alpha-linolenic acid (ALA) have been associated with the risk of developing CD^6,9–11^.

The potential role of fatty acids and adipose tissue in inflammation has increased interest in fatty acid profiling and manipulation in CD. The inflammatory state in IBD is associated with increased eicosanoids including prostaglandin E_2_ and leukotriene B_4_^12,13^, which are derived from the metabolism of the PUFA arachidonic acid (AA). Fatty acids and eicosanoids are implicated in multiple signaling cascades involved in inflammation including vascular permeability, edema, and tissue damage^14–17^. In addition, the n-3 fatty acids eicosapentaenoic acid (EPA) and docosahexaenoic acid (DHA) have anti-inflammatory properties^18,19^. Resolvins and protectins, which are synthesized from DHA and EPA, are anti-inflammatory mediators that promote resolution of inflammation by reducing neutrophil infiltration and attenuating production of pro-inflammatory cytokines including tumor necrosis factor (TNF)^20–22^. In addition, adipose tissue itself has been implicated in the inflammatory state via secretion of inflammatory cytokines (such as TNF-α, IL-1β, IL-6, IL-8, and IL-10) and adipocyte-derived paracrine mediators, termed adipokines (such as leptin, resistin, adiponectin, adipsin, and plasminogen activator inhibitor-1 (PAI-1))^23–25^.

It has been hypothesized that IBD patients would have decreased blood and tissue PUFA, specifically n-3 PUFA, due to the inflammatory state. However, a prior study of plasma PUFA in IBD showed a significantly higher fraction of the n-3 PUFAs, ALA and DHA, and lower n-6 PUFA in active IBD versus controls^26,27^. These alterations persisted in inactive CD^27^. In addition, a smaller study showed no significant differences in plasma phospholipids, but did show alterations in PUFA in erythrocyte membrane phospholipids^28^. There has been interest in the effects of dietary supplementation of n-3 fatty acids in CD, but the results of trials have been inconsistent and largely inconclusive^29–32^. These studies, however, did not take into account many complex interacting factors in this population, including baseline diet, genetics, or measurable serum fatty acids.

We have previously shown in a prospective cohort, that UC patients have significantly lower serum % saturated fatty acids (SFA) and % AA, but a higher % monounsaturated fatty acids (MUFA), (EPA + DHA)/AA ratio, % oleic acid, and % LA versus controls^33^. While these alterations did not correlate with serum cytokines, the serum % SFA directly correlated and serum total % PUFA, EPA, and docosapentaenoic acid (DPA) inversely correlated with pro-inflammatory cytokines in active UC colon tissue^33^.

We hypothesize that n-6 PUFA play a key role in modifying pro-inflammatory cytokines and disease status in CD and, therefore, serum levels of PUFA would correlate inflammatory cytokines and disease activity in CD. The current study aims to (1) investigate serum cytokine/adipokine levels, serum fatty acid composition patterns, and dietary fatty acid intake in CD and controls, and (2) determine if there is an association of serum fatty acid composition and serum cytokines or adipokines.

## Results

### Patient Characteristics

In total, 116 CD patients and 27 non-IBD controls were included in the cohort. Table 1 shows the patient characteristics. There was no significant difference in age, gender distribution, body mass index (BMI), or smoking status in CD versus controls. The majority of CD patients were on at least one disease-specific therapy (n = 106, 91.4%), with the majority being on an anti-TNF-α agent (n = 76, 65.5%) either alone (n = 42, 36.2%) or in combination with an immunomodulator (n = 34, 29.3%). CD patients had an average of 11.7 years of disease and approximately half (n = 63, 54.3%) of CD patients had a prior CD related surgery.

**Table 1:** Patient characteristics by diagnosis.

	Control n = 27	CD n = 116
Age, mean (SD)	45.2 (12.0)	40.6 (13.0)
Male Gender, n (%)	10 (37.0%)	58 (50.0%)
Body Mass Index, mean (SD)	28.0 (5.8)	28.0 (6.6)
Tobacco Use, n (%)	2 (7.4%)	15 (12.9%)
Any IBD Therapy, n (%)	—	106 (91.4%)
5-ASA alone, n (%)	—	6 (5.2%)
Corticosteroid use, n (%)	—	12 (10.3%)
Immunomodulator alone, n (%)	—	14 (12.1%)
Anti-TNF-α alone, n (%)	—	42 (36.2%)
Anti-TNF-α + Immunomodulator Combination Therapy, n (%)	—	34 (29.3%)
Vedolizumab, n (%)	—	1 (0.9%)
Ustekinumab, n (%)	—	6 (5.2%)
IBD Related Surgery, n (%)	—	63 (54.3%)
Ileo-colic Resection, n (%)	—	30 (25.9%)
Small Bowel Resection, n (%)	—	8 (6.9%)
Colectomy, n (%)	—	3 (0.9%)
Multiple Surgeries, n (%)	—	22 (19.0%)
Stricturing Phenotype, n (%)	—	50 (43.1%)
Penetrating Phenotype, n (%)	—	35 (30.2%)
Perianal Disease, n (%)	—	38 (32.8%)
Years of Crohn’s Disease, mean (SD)	—	11.67 (9.92)
Small Bowel Disease, n (%)	—	44 (37.9%)
Large Bowel Disease, n (%)	—	15 (12.9%)
Small and Large Bowel Disease, n (%)	—	15 (12.9%)
Upper GI Involvement, n (%)	—	9 (7.8%)

Age and Body Mass Index (BMI) are represented by mean (SD) and were analyzed by the Mann-Whitney U test. Categorical variables are represented by number (%) and were analyzed using the Pearson’s χ^2^ test. Age, BMI, gender, and tobacco use were not significantly different from control.

### Serum Fatty Acids Are Altered in CD Versus Control

Serum fatty acid analysis showed increased percentages of MUFA, dihomo-gamma-linolenic acid (DGLA), EPA, DPA, and oleic acid, but decreased AA in the serum of CD versus controls (Table 2). Similar patterns were seen when we stratified CD patients based on clinically active or inactive disease. (Supplemental Table 1).

**Table 2:** Serum fatty acids are altered in CD compared with control subjects.

	Control n = 27	CD n = 116
Total (μg/mL)	1894.12 ± 306.57	2081.70 ± 549.47
% SFA	43.29 ± 1.23	43.12 ± 1.75
% PUFA	47.04 ± 1.63	46.58 ± 2.16
% MUFA	9.67 ± 1.44	10.30 ± 1.40*
% n-3	4.81 ± 1.20	5.30 ± 1.72
% n-6	42.23 ± 1.88	41.28 ± 2.42
n-3/n-6	0.11 ± 0.03	0.13 ± 0.05
(EPA + DHA)/AA	0.28 ± 0.10	0.35 ± 0.15
% Arachidonic Acid	13.44 ± 2.21	12.04 ± 1.94**
% DGLA	3.16 ± 0.65	3.69 ± 0.81**
% ALA	0.27 ± 0.08	0.27 ± 0.01
% Linoleic Acid	24.79 ± 2.32	24.63 ± 3.23
% Oleic Acid	8.09 ± 1.35	8.55 ± 1.22*
% EPA	0.71 ± 0.46	1.16 ± 0.72**
% DPA	0.92 ± 0.17	1.03 ± 0.24*
% DHA	3.01 ± 0.79	2.93 ± 1.16

Comparisons between the CD and control groups were assessed by the Mann-Whitney U test. Data is presented by mean ± standard deviation. *p < 0.05 and **p < 0.01 versus control. SFA = Saturated Fatty Acids; MUFA = Monounsaturated Fatty Acids; PUFA = Polyunsaturated Fatty Acids; EPA = Eicosapentaenoic acid; DPA = Docosapentaenoic acid; DHA = Docosahexaenoic acid.

### Dietary Intake

Of 63 CD and 10 controls who were recruited after November 2016, 38 CD (60.3%) and 9 controls (90.0%) completed the dietary intake assessments. The first of three dietary assessment calls were attempted within 7 days of serum collection. However, the median time between serum collection and completion of all dietary interviews was 63 (range 12–96) days. Missing patients (35.6%) could not be contacted by telephone or email despite multiple attempts. CD subjects had higher total energy (kcal) intake (1933.27 ± 686.28 vs. 1416.48 ± 443.48 kcal, p = 0.045), but there were no significant differences in total fat or PUFA intake versus controls (Table 3). Total dietary fat intake correlated with total serum phospholipids in CD (R = 0.4253, p = 0.02), but not in controls, and there were no other significant correlations between individual serum fatty acid levels and dietary fatty acid intake in either CD or controls (data not shown).

**Table 3:** Dietary intake of fat in CD and control subjects.

	Control n = 9	CD n = 38	p-value
Total Energy (kcal)	1416.48 ± 443.48	1933.27 ± 686.28	0.045
Total Fat (g)	60.13 ± 21.78	75.48 ± 30.60	0.234
Total SFA (g)	22.06 ± 10.14	26.10 ± 10.47	0.256
Total MUFA (g)	20.95 ± 7.16	26.89 ± 12.50	0.160
Total PUFA (g)	12.39 ± 5.23	15.96 ± 7.86	0.245
Total n-3 (g)	1.17 ± 0.45	1.50 ± 0.76	0.330
Total n-6 (g)	11.23 ± 4.90	14.47 ± 7.17	0.245
Oleic Acid (g)	19.70 ± 6.88	25.19 ± 11.82	0.234
Linoleic Acid (g)	10.99 ± 4.90	14.16 ± 7.11	0.280
α-linolenic Acid (g)	1.12 ± 0.45	1.43 ± 0.77	0.358
Arachidonic Acid (g)	0.11 ± 0.05	0.14 ± 0.08	0.358
EPA (g)	0.01 ± 0.01	0.02 ± 0.01	0.111
DPA (g)	0.01 ± 0.01	0.02 ± 0.01	0.164
DHA (g)	0.03 ± 0.18	0.04 ± 0.03	0.256

Data is expressed as the mean ± SD of average daily intake. Mann-Whitney U test was performed. SFA = Saturated Fatty Acids; MUFA = Monounsaturated Fatty Acids; PUFA = Polyunsaturated Fatty Acids; EPA = Eicosapentaenoic acid; DPA = Docosapentaenoic acid; DHA = Docosahexaenoic acid.

### Relation of Serum Fatty Acids to Demographics in CD

Some of the demographic characteristics were associated with variation in fatty acid levels in CD patients. Total phospholipids (R = 0.3284, p < 0.001), % n-3 (R = 0.2986, p = 0.001), n-3/n-6 ratio (R = 0.2916, p = 0.002), and (EPA + DHA)/AA ratio (R = 0.2574, p = 0.005) correlated with age with CD patients. Total phospholipids were lower in male compared with female CD patients (1937.2 ± 443.0 vs. 2226.2 ± 591.9 µg/mL, p = 0.005), whereas, % linoleic acid was higher in male compared with female CD patients (25.25 ± 3.15 vs. 24.01 ± 3.21, p = 0.02). Body Mass Index (BMI) correlated with % MUFA (R = 0.3032, p = 0.001) and oleic acid (R = 0.2887, p = 0.002) in CD. Years of CD correlated with total phospholipids (R = 0.2051, p = 0.03) and % EPA (R = 0.2386, p = 0.01), but inversely correlated with linoleic acid (R = −0.1939, p = 0.04). There were no significant differences in lipids in CD smokers vs. non-smokers (data not shown). We were not powered to show differences in these variables in our control group, but prior studies have shown similar trends based on gender, age, and BMI in healthy individuals suggesting these observations are not CD specific^34–36^. In addition, these demographic variables were not different between our CD and control subjects (Table 1) making it unlikely that these relationships are driving the differences seen in the serum fatty acids between the two groups.

When CD subjects were categorized by medication use, only those on anti-TNF-α agents compared with those receiving no disease-specific medications showed significantly different changes in pairwise comparisons between medication subgroups (Supplemental Table 2). In addition, the subgroup of CD patients on no disease-specific medications had fatty acid alterations compared to control that were similar in magnitude and direction to those observed in active CD (Supplemental Table 1), whereas, those on various medications did not consistently trend in the same direction.

### Serum Fatty Acids Correlate with Clinical Disease Activity

**Figure 1:**
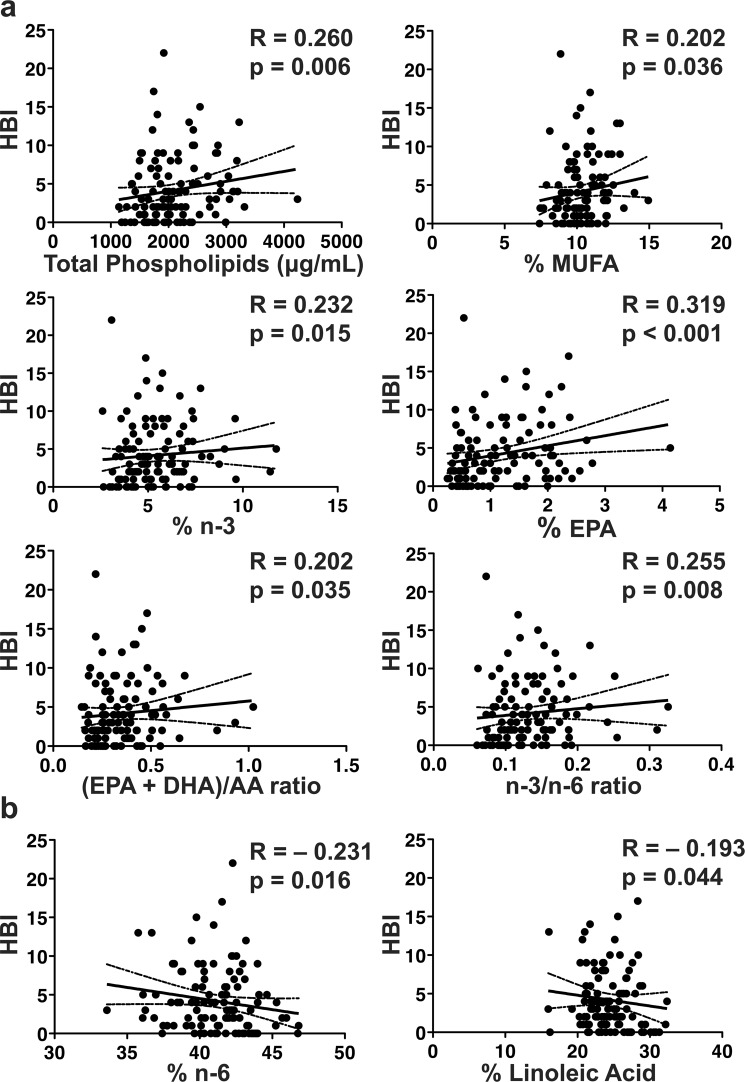
Clinical disease activity correlates with serum fatty acids. Clinical disease activity correlates directly with (**a**) total phospholipids, % MUFA, % n-3, % EPA, (EPA + DHA)/AA ratio, and n-3/n-6 ratio, but inversely correlates with (**b**) % n-6 and linoleic acid. Clinical disease activity by HBI shown as a continuous variable on the Y-axis. The spearman rho (R) is shown with corresponding p-value. The solid line represents the line of best fit with 95% confidence intervals (dashed lines). *n* = 111 for CD patients.

To evaluate the relationship of clinical disease activity to serum fatty acids, we correlated serum fatty acids to HBI in 111 CD subjects with both serum fatty acid and HBI measurements. The total phospholipids, total % MUFA, total % n-3, % EPA, (EPA + DHA)/AA ratio, and n-3/n-6 were all directly correlated with clinical disease activity (Fig. 1a). The total % n-6 and % linoleic acid were inversely correlated with clinical disease activity in CD subjects (Fig. 1b). Correlations with clinical disease activity were similar in direction to those with duration of CD as outlined above, suggesting these alterations may be influenced by disease duration and severity.

### Multiple Serum Cytokines and Adipokines Are Altered in CD versus Controls

After identifying correlations between serum fatty acids and clinical disease activity, we compared the serum fatty acids to serum inflammatory mediators. First, we measured serum cytokines and adipokines in CD and controls to determine those that were significantly altered in CD subjects. We have previously shown that only 2 cytokines, CCL11 (also known as Eotaxin-1) and G-CSF were significantly increased in UC patient serum versus controls^33,37^. We have now found that 6 cytokines, out of 32 assessed, were significantly increased in the serum of CD patients versus controls with a false discovery rate (q-value) of <0.05 to adjust for multiple comparisons in our multiplex assay (corresponding to a maximum p-value 0.012) including: CCL11, GM-CSF, GRO (also known as CXCL1), IL-17A, VEGF, and IL-7 (Table 4). Three adipokines (out of 8 assessed), lipocalin-2 (also known as NGAL), resistin, and hepatocyte growth factor (HGF), were significantly increased in CD versus controls (Table 4). When stratified by disease activity, the majority of these remained significantly different after false discovery rate correction in both active and inactive CD versus controls (Supplemental Table 3).

**Table 4:** Serum cytokines and adipokines in CD vs. control subjects.

	Control n = 27	CD n = 114	p-value	q-value
GM-CSF (pg/mL)	3.5 ± 3.2	21.3 ± 24.8	<0.001	0.010
Lipocalin-2 (ng/mL)	287.8 ± 143.6	480.2 ± 261.5	<0.001	0.010
CCL11 (pg/mL)	140.3 ± 86.8	246.3 ± 164.4	<0.001	0.010
GRO (pg/mL)	899.5 ± 428.0	1274.2 ± 863.4	0.005	0.029
VEGF (pg/mL)	139.8 ± 106.7	303.1 ± 258.6	0.006	0.029
HGF (pg/mL)	460.8 ± 235.7	687.2 ± 427.9	0.006	0.029
Resistin (ng/mL)	33.0 ± 12.9	45.0 ± 21.2	0.005	0.029
IL-17A (pg/mL)	1.8 ± 1.6	9.1 ± 15.2	0.009	0.037
IL-7 (pg/mL)	2.7 ± 2.9	6.4 ± 6.6	0.012	0.045
G-CSF (pg/mL)	16.8 ± 19.8	36.4 ± 39.4	0.020	0.067
IFN-γ (pg/mL)	10.2 ± 10.6	35.1 ± 41.1	0.023	0.070
IL-5 (pg/mL)	2.4 ± 2.8	14.3 ± 22.6	0.035	0.078
IL-8 (pg/mL)	8.9 ± 7.9	17.0 ± 20.3	0.032	0.078
IL-9 (pg/mL)	1.1 ± 0.7	4.0 ± 4.1	0.035	0.078
IL-12p40 (pg/mL)	25.2 ± 26.0	119.1 ± 158.7	0.037	0.078
Adipsin (ng/mL)	3016.5 ± 610.4	2794.5 ± 1002.0	0.029	0.078
Adiponectin (ng/mL)	36290.8 ± 34202.6	30218.8 ± 39636.7	0.045	0.089
MIP-1α (pg/mL)	12.0 ± 6.6	52.4 ± 67.5	0.049	0.090
MIP-1β (pg/mL)	20.8 ± 12.7	38.9 ± 39.2	0.058	0.090
IL-15 (pg/mL)	2.4 ± 1.3	8.1 ± 10.4	0.057	0.090
IL-10 (pg/mL)	3.2 ± 2.4	15.4 ± 21.3	0.052	0.090
IFNA2 (pg/mL)	69.3 ± 40.0	31.8 ± 30.8	0.059	0.090
FGF-2 (pg/mL)	60.3 ± 48.3	79.4 ± 52.3	0.100	0.132
TGF-α (pg/mL)	10.4 ± 20.1	4.8 ± 5.8	0.103	0.132
IL-1α (pg/mL)	38.3 ± 49.9	110.8 ± 116.1	0.108	0.132
IL-6 (pg/mL)	2.3 ± 2.6	6.4 ± 7.9	0.110	0.132
MDC (pg/mL)	971.2 ± 357.8	1131.6 ± 475.7	0.110	0.132
PAI-1 Total (ng/mL)	69.2 ± 22.5	77.5 ± 25.4	0.101	0.132
MCP-1 (pg/mL)	427.4 ± 120.9	510.2 ± 254.9	0.238	0.276
MCP3 (pg/mL)	120.8 ± 92.3	294.4 ± 416.2	0.254	0.284
IP-10 (pg/mL)	361.2 ± 124.4	373.3 ± 269.4	0.274	0.297
NGF (pg/mL)	1.9 ± 1.5	4.0 ± 5.9	0.292	0.307
EGF (pg/mL)	76.4 ± 45.5	93.6 ± 61.4	0.311	0.317
TNF-α (pg/mL)	7.6 ± 3.1	12.8 ± 25.7	0.370	0.367
TNF-β (pg/mL)	65.1 ± 64.5	169.1 ± 251.6	0.452	0.434
SCD40L (pg/mL)	6088.7 ± 4605.2	4923.9 ± 2966.3	0.506	0.472
IL-13 (pg/mL)	34.2 ± 30.1	93.3 ± 142.1	0.617	0.559
Leptin (pg/mL)	19081.1 ± 14513.8	18957.0 ± 18409.4	0.632	0.559
Fractalkine (pg/mL)	160.6 ± 136.3	217.1 ± 289.5	0.877	0.737
IL-1ra (pg/mL)	43.3 ± 51.0	103.4 ± 231.4	0.863	0.737

Data are shown as mean ± SD. P-values are calculated with a Mann-Whitney U test and q-values by the Benjamini and Hochberg method where 0.05 was considered significant. Two samples were missing cytokine/adipokine measurements.

Given that 65.5% of our CD patients were on anti-TNF-α therapy, either alone or in combination with an immunomodulator, we assessed what effects this had on serum cytokine levels, including TNF-α. After removing subjects who were receiving an anti-TNF-α agent (n = 76) at the time of serum collection, serum TNF-α levels were significantly higher in CD versus controls (13.47 ± 10.85 pg/mL vs. 7.56 ± 3.06 pg/mL, q = 0.01) (Supplemental Table 4). Several additional serum cytokines and adipokines showed significant differences versus controls only after exclusion of subjects on an anti-TNF-α agent, suggesting that anti-TNF-α therapy may alter levels of cytokines other than TNF-α (Supplemental Table 4).

### Serum % n-3 and % n-6 Fatty Acids Correlate with Pro-Inflammatory Cytokines in CD

To capture a range of cytokines that are relevant to CD, we compared serum fatty acid levels to the 18 serum cytokines/adipokines that were significantly or borderline significantly (p-value of < 0.05 regardless of FDR) increased in CD (Table 4), as well as TNF-α, since this was significant in subjects not on anti-TNF-α agents.

**Figure 2:**
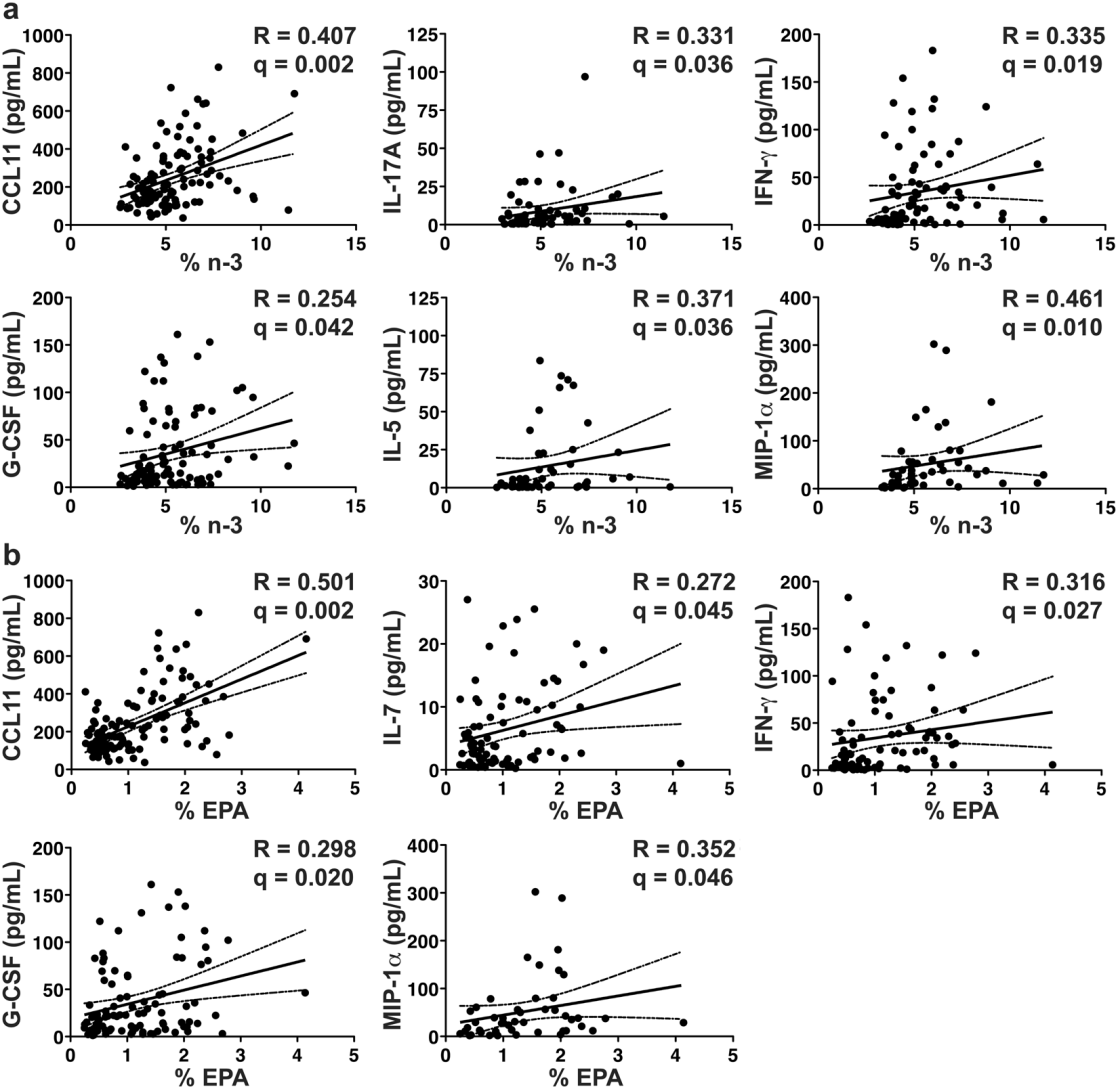
Serum % n-3 fatty acids directly correlate with serum pro-inflammatory cytokines. Serum % n-3 (**a**) correlates directly to CCL11, IL-17A, IFN-γ, G-CSF, IL-5, and MIP-1α. Serum % EPA (**b**) correlates directly to CCL11, IL-7, IFN-γ, G-CSF, and MIP-1α. The Spearman rho (R) is shown with the corresponding p-value. The solid line represents the line of best fit with 95% confidence intervals (dashed lines). Serum % n-3 did not correlate with any cytokines/adipokines in controls. *n* = 114 for CD patients.

After again correcting for multiple cytokine comparisons with a false discovery rate of 0.05 (q-value), the total serum % n-3 fatty acids were directly correlated with CCL11, IL-17A, IFN-γ, G-CSF, IL-5, and MIP-1α (also known as CCL3) (Fig. 2a). Similarly, the % EPA, an n-3 fatty acid, was directly correlated with CCL11, IL-7, IFN-γ, G-CSF, and MIP-1α (Fig. 2b). In general, correlations with % EPA were stronger than those with total % n-3, suggesting that EPA may be a key driver of this relationship.

**Figure 3:**
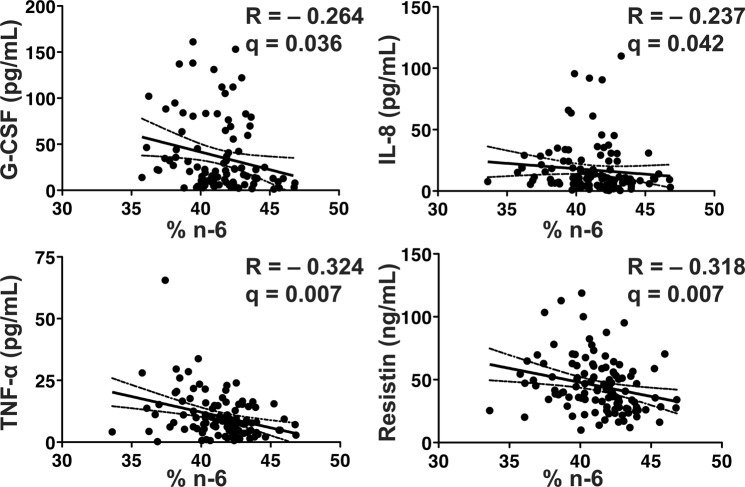
Serum % n-6 inversely correlates with serum pro-inflammatory cytokines. Serum % n-6 inversely correlates with G-CSF, IL-8, TNF-α, and resistin. The Spearman rho (R) is shown with the corresponding p-value. The solid line represents the line of best fit with 95% confidence intervals (dashed lines). Serum % n-6 did not correlate with any cytokines/adipokines in controls. *n* = 114 for CD patients.

Serum % n-6 fatty acids were inversely correlated with G-CSF, IL-8, TNF-α, as well as the pro-inflammatory adipokine resistin (Fig. 3). Neither the % AA or % DGLA were significantly correlated with any of the evaluated pro-inflammatory cytokines (data not shown). There were no significant correlations between % n-3 or % n-6 fatty acids and the serum cytokines/adipokines assessed in controls (n = 27; data not shown). Taken together our data suggest that serum % n-3 fatty acids, rather than % n-6 fatty acids, are associated with inflammatory activity in CD.

## Discussion

This study shows a detailed description of serum fatty acids in relation to serum cytokines and adipokines in CD. We demonstrate that serum % n-3 fatty acids tend to be increased, whereas, serum % n-6 fatty acids are decreased in CD versus controls. In patients with CD, there are also notable differences in serum pro-inflammatory cytokines and adipokines compared to controls. In addition, we describe that serum % n-3 fatty acids directly correlated with serum cytokines and clinical disease activity, whereas serum % n-6 fatty acids inversely correlated with pro-inflammatory cytokines and clinical disease activity.

A similar pattern of increased n-3 and decreased n-6 fatty acids has been shown previously in CD versus control^26,27,38^. We did not, however, observe the differences in ALA seen previously^26^. Our findings are consistent with the pattern reported previously in our UC cohort showing increased levels of serum MUFA, linoleic acid, oleic acid, EPA, and DPA, but decreased AA in UC versus controls^33^. In CD, however, we did not observe differences in SFA, which were notable in our prior UC cohort^33^. In addition, we have recently shown that lipid, amino acid, and energy metabolism is altered in CD compared to UC and control patients using metabolomic profiling^39^. This pattern suggests that increased PUFA biosynthesis may coexist with increased fatty acid utilization in CD. There is existing evidence of increased synthesis of AA-derived eicosanoids in the intestinal mucosa of active IBD patients, which could account for increased utilization and, therefore, a decrease in serum n-6 fatty acids^40,41^. The n-6 fatty acid DGLA, in contrast to AA, was increased in CD. DGLA is metabolized by cyclooxygenase 1 and 2 (prostaglandin G/H synthase 1 and 2), as well as arachidonate 15-lipoxygenase, into prostaglandins and anti-inflammatory eicosanoids with the ability to antagonize synthesis of AA-derived pro-inflammatory eicosanoids^42^. It is notable, however, that one prior study showed decreased, rather than increased DGLA in the plasma of patients with clinically active CD^26^, but another study of inactive CD versus control did not show any significant alterations in DGLA^27^. Clinical disease activity may play a role in the differences in DGLA levels noted between our study and others.

To relate our findings to objective measures of the inflammatory state, we noted that fatty acids correlated with cytokine and adipokine levels. We noted multiple significant changes in both serum cytokines and adipokines in CD versus controls (Table 4). Interestingly, our prior study in UC revealed only 2 significantly increased serum cytokines compared with controls, perhaps due to the less systemic nature of the disease process^33^. The direct correlation of % n-3 and inverse correlation of % n-6 with pro-inflammatory cytokines, further supports the hypothesis that there is a dysregulation of PUFA in CD that is more pronounced with increasing disease activity. This provides support for manipulation of serum fatty acid composition through diet or medication to modify cytokine production.

Prior interventional studies have focused only on supplementing n-3 fatty acids. However, our data suggests that circulating levels of % n-3 fatty acids are elevated in this population and support the observation that oral n-3 supplementation alone is unlikely to counteract the dysregulated PUFA metabolism in CD. Future interventions may need to focus on normalizing the n-3/n-6 ratio or focus on further understanding utilization of PUFA in this population. Interestingly, prior studies have shown that susceptibility polymorphisms in genes associated with PUFA metabolism (*FADS1*, *FADS2*, and *CYP4F3*) in the setting of increased dietary n-6/n-3 intake can increase the risk of developing IBD^6,11^. It is hypothesized that carriers of susceptibility alleles may metabolize n-3 fatty acids less efficiently into anti-inflammatory resolvins and protectins, and may divert PUFA metabolism toward the n-6 pathways.

Overall, dietary intake was unchanged in CD versus controls with the exception of increased caloric consumption in CD subjects (Table 3). This increase in caloric intake has been shown previously^28^, however, we did not show any alterations in fat intake that have been suggested in prior epidemiologic studies. Thus, we cannot attribute the observed changes to differences in dietary intake, however, our dietary intake analysis was limited by low response rates and subsequently a small sample size.

The current study is limited by the lack of tissue fatty acids or cytokines. Several studies have shown alterations in PUFA in inflamed tissue of IBD patients including increases in AA and decreases in EPA^41,43^. In our prior study of UC subjects, serum n-3 PUFA were inversely correlated with multiple tissue cytokines, but not with serum cytokines^33^. There may be variation in the metabolism of fatty acids in inflamed tissue compared to serum free fatty acids. Supporting this, a recent study showed altered metabolism of AA and DHA in inflamed colonic tissues of UC patients when compared to controls or resolving UC^44^. However, given the more systemic inflammatory pattern in CD, as evidenced by the large number of increased serum cytokines, our current study retains substantial relevance. In addition, we are limited by the lack of treatment naïve subjects. The use of anti-TNF-α agents seems to lessen the differences between CD and controls in several cytokines other than TNF-α (Supplemental Table 4) and may have decreased the strength of the observed correlations. Our patient population had high rates of individuals who were overweight and obese in both the CD and control groups. The mean BMI was in the overweight category (BMI > 25) in both groups (Table 1). While there was no difference in the groups, the prevalence of overweight and obese individuals may alter the absolute levels of adipokines and fatty acids as compared to a population with normal body mass. In addition, this study used measures of clinical disease activity and future studies are needed to compare these findings to endoscopic or histologic markers of disease activity.

In summary, patients in our CD cohort had altered serum fatty acid profiles that are not fully explained by alterations in dietary intake. Our data suggests that there may be altered utilization/metabolism of n-3 fatty acids resulting in their accumulation in the serum, accompanied by increased n-6 utilization and pro-inflammatory cytokine production. Correcting this n-3/n-6 ratio may be a potential therapeutic strategy in IBD, in particular CD.

## Materials and Methods

### Study Subjects

Samples were obtained from two prospective cohorts of CD and control subjects. CD subjects were recruited from the Vanderbilt University Medical Center IBD center from November 2014 to May 2017. Control serum was obtained from healthy adult volunteers without a history of IBD. The study protocol was approved by the Vanderbilt University Medical Center Institutional Review Board. All methods were performed in accordance with relevant guidelines and regulations for human subjects research. Written informed consent was obtained for analysis of demographics, medical and dietary histories, as well as serum samples as part of two separate prospective studies. Subjects were adult patients (>18 years old) with CD confirmed by standard endoscopic, histologic, and radiologic assessment. Participants were excluded if they (1) were pregnant, (2) had a coagulopathy or bleeding disorder, (3) had renal or hepatic impairment, (4) had a history of organ transplantation, or (5) had an unstable clinical condition (bleeding, infection, intestinal obstruction, *etc*.). After collection, study serum was processed within 1 hour, snap frozen on dry ice, and then stored at −80 °C. Demographic and clinical data including clinical disease activity as assessed by the Harvey Bradshaw Index (HBI), current medication use, and disease distribution/characteristics were obtained for each CD subject at the time of serum collection. Clinically active disease was defined by a HBI > 4 and clinically inactive disease a HBI ≤ 4.

### Serum Fatty Acids

Serum fatty acids were analyzed by the Vanderbilt Hormone and Analytical Services Core. Serum lipids were extracted using the Folch *et al*. method as previously described^33,45^. The extracts were filtered, and lipids recovered in the chloroform phase. Individual lipid classes were separated by thin layer chromatography using Silica Gel 60A plates developed in petroleum ether, ethyl ether, acetic acid (80:20:1), and visualized by rhodamine 6G. Phospholipids, diglycerides, triglycerides, and cholesteryl esters were scraped from the plates and methylated using BF3/methanol as Morrison and Smith described^46^. The methylated fatty acids were extracted and analyzed by gas chromatography. Gas chromatographic analyses were carried out on an Agilent 7890A gas chromatograph equipped with flame ionization detectors, and a capillary column (SP2380, 0.25 mm × 30 m, 0.25 μm film, Supelco, Bellefonte, PA). The carrier gas was helium. The oven temperature was programmed from 160 °C to 230 °C at 4 °C/min. Fatty acid methyl esters were identified by comparing the retention times to those of known standards. Inclusion of lipid standards with odd chain fatty acids permitted quantitation of the lipid amounts in the sample. Dipentadecanoyl phosphatidylcholine (C15:0), diheptadecanoin (C17:0), trieicosenoin (C20:1), and cholesteryl eicosenoate (C20:1) were used as standards. Output is expressed as percent of total phospholipid composition.

### Serum Cytokines and Adipokines

Serum cytokines were measures as previously described using Luminex technology with Milliplex MAP (Millipore, Billerica, MA) multiplex magnetic bead-based antibody detection kits according to the manufacturer’s protocols and assayed on a FLEXMAP 3D machine^37^. Serum samples were analyzed with a pre-mixed 38 cytokine analyte kit. Samples were run in duplicate and quality control samples were run across plates. Analytes that were consistently below the lower limit of detection (>70% of the values) were excluded from further analysis (including IL-1β, FLT-3L, IL-12p70, IL-2, IL-3, and IL-4). In addition, we assessed the following serum adipokines using custom kits: lipocalin-2 (also known as NGAL), resistin, adipsin, adiponectin, plasminogen activator inhibitor-1 (PAI-1), nerve growth factor (NGF), leptin, and hepatocyte growth factor (HGF) using the same methods according to the manufacturer’s protocols.

### Dietary Intake

Subjects recruited after November 2016 (63 CD and 10 controls) underwent 3 separate 24-hour dietary recalls to assess dietary intake. Participants before November 2016 were consented on a separate study and could not be re-contacted for dietary intake. A trained research coordinator conducted 3 separate interviews focused on food, snack, and beverage intake during the previous 24-hours using the Nutrient Data System for Research software (U. of MN) as previously described^33,47^. The first interview was attempted within 7 days of serum collection. The second and third interviews were based on patient availability with attempts made to sample both weekdays and weekends. Subjects who completed at least 2 of the 3 dietary recalls were included in the final analysis. The average daily intake is reported in grams.

### Statistical Analysis

Data are expressed as mean ± standard deviation (SD) for continuous variables or number (%) for categorical variables. Outlier testing was done on all data using the Grubbs method^48^. For 2 group comparisons, a Mann-Whitney U test was completed. Data with more than 2 groups was analyzed with a Kruskal-Wallis test and if p < 0.05, then pairwise comparisons were made with the Mann-Whitney U test. Categorical data was analyzed with a Pearson’s χ^2^ test. Correlations were determined using Spearman’s rank correlation. To account for multiple comparisons when analyzing multiple cytokines, the p-value was adjusted for a false discovery rate using the Benjamini and Hochberg method^49^. A false discovery rate (or q-value) of 0.05 was considered significant. We considered cytokines with an uncorrected p-value < 0.05 that did not reach the q-value < 0.05 to be borderline significant and these were included in comparisons with fatty acids. Statistical analyses were completed with STATA v. 14.2 (College Station, TX) and GraphPad Prism 7 (La Jolla, CA).

## Supplementary information

Supplemental Materials

## Data Availability

The dataset utilized for this study was prospectively collected from consented patients. The database has not been made publicly available due to the presence of protected health information (e.g. dates of encounters to track time between dietary recalls and encounter for blood draw).
