# Supplementary material for: Serum Polyunsaturated Fatty Acids Correlate with Serum Cytokines and Clinical Disease Activity in Crohn’s Disease
Source: Sci Rep. 2019 Feb 27;9:2882. doi: 10.1038/s41598-019-39232-z (PMC6393448; doi:10.1038/s41598-019-39232-z)
Supplement: Supplementary file 1 — Supplemental Materials [file 41598_2019_39232_MOESM1_ESM.docx]

**SUPPLEMENTAL MATERIAL**

**Serum Polyunsaturated Fatty Acids Correlate with Serum Cytokines and Clinical Disease Activity in Crohn’s Disease**

Elizabeth A. Scoville MD MSCI^1^, Margaret M. Allaman BS^1^, Dawn W. Adams MD MS^1,4^, Amy K. Motley BS^1^, Shannon C. Peyton LPN^1^, Sarah L. Ferguson RD^1^, Sara N. Horst MD MPH^1^, Christopher S. Williams MD PhD^1,3,4,5,6^, Dawn B. Beaulieu MD^1^, David A. Schwartz MD^1^, Keith T. Wilson MD^1,2,3,4,6^, Lori A. Coburn MD*^1,3,4^

^1^Division of Gastroenterology, Hepatology, and Nutrition, Department of Medicine, Vanderbilt University Medical Center, Nashville, TN, USA

^2^Department of Pathology, Microbiology, and Immunology, Vanderbilt University Medical Center, Nashville, TN, USA

^3^Vanderbilt Center for Mucosal Inflammation and Cancer, Department of Medicine, Vanderbilt University Medical Center, Nashville, TN, USA

^4^Veterans Affairs Tennessee Valley Healthcare System, Nashville, TN, USA

^5^Vanderbilt Center for Stem Cell Biology, Vanderbilt University Medical Center, Nashville, TN, USA

^6^Vanderbilt Ingram Cancer Center, Vanderbilt University Medical Center, Nashville, TN, USA

**CORRESPONDENCE: ***Dr. Lori A Coburn, Vanderbilt University Medical Center, Division of Gastroenterology, 2215B Garland Ave., 1030C MRB IV, Nashville, TN, USA 37232

Phone: 615-875-4222; Fax: 615-343-6229, E-mail: [lori.coburn@vumc.org](mailto:lori.coburn@vumc.org)

|  | **Control**  **n = 27** | **Inactive**  **n = 72** | **Active**  **n = 39** |
| --- | --- | --- | --- |
| **Total (μg/mL)** | 1894.12 ± 306.57 | 2052.70 ± 571.45 | 2173.27 ± 521.69* |
| **% SFA** | 43.29 ± 1.23 | 43.23 ± 1.84 | 43.89 ± 1.65 |
| **% PUFA** | 47.04 ± 1.63 | 46.61 ± 2.30 | 46.51 ± 2.01 |
| **% MUFA** | 9.67 ± 1.44 | 10.16 ± 1.47 | 10.61 ± 1.28** |
| **% n-3** | 4.81 ± 1.20 | 5.19 ± 1.62 | 5.66 ± 1.88 |
| **% n-6** | 42.23 ± 1.88 | 41.42 ± 2.55 | 40.85 ± 2.20* |
| **n-3/n-6** | 0.11 ± 0.03 | 0.13 ± 0.05 | 0.14 ± 0.01* |
| **(EPA+DHA)/AA** | 0.28 ± 0.10 | 0.34 ± 0.15 | 0.38 ± 0.17* |
| **% Arachidonic Acid** | 13.44 ± 2.21 | 12.20 ± 1.91** | 11.85 ± 1.94** |
| **% DGLA** | 3.16 ± 0.65 | 3.68 ± 0.83** | 3.77 ± 0.80** |
| **% ALA** | 0.27 ± 0.08 | 0.27 ± 0.10 | 0.28 ± 0.10 |
| **% Linoleic Acid** | 24.79 ± 2.32 | 24.60 ± 3.29 | 24.29 ± 2.93 |
| **% Oleic Acid** | 8.09 ± 1.35 | 8.43 ± 1.27 | 8.80 ± 1.12* |
| **% EPA** | 0.71 ± 0.46 | 1.05 ± 0.67* | 1.38 ± 0.78***# |
| **% DPA** | 0.92 ± 0.17 | 1.03 ± 0.24* | 1.05 ± 0.25* |
| **% DHA** | 3.01 ± 0.79 | 2.95 ± 1.15 | 2.99 ± 1.23 |

Supplemental Table 1. **Serum Fatty Acids are Altered in CD by Clinical Disease Activity Compared with Control Subjects.** The three groups were assessed by the Kruskal-Wallis test and, if p < 0.05, were followed by the Mann-Whitney U test to compare individual group pairs. Clinically active disease was defined by a HBI > 4 and clinically inactive disease a HBI ≤ 4. *p < 0.05, **p < 0.01, and ***p < 0.001 versus control. #p < 0.05 vs. inactive CD. Of 116 CD subjects with fatty acid measurements, five patients were missing HBI values.

|  | **Control**  **(n = 27)** | **CD –**  **No CD Therapy**  **(n = 10)** | **CD –**  **Immunomodulator only**  **(n = 14)** | **CD –**  **anti-TNF-α only**  **(n = 42)** | **CD –**  **anti-TNF-α + Immunomodulator**  **(n = 34)** |
| --- | --- | --- | --- | --- | --- |
| **Total (μg/mL)** | 1894.12 ± 306.57 | 2202.32 ± 634.71 | 2207.48 ± 753.39 | 1939.52 ± 528.39 | 2231.69 ± 514.14 |
| **% SFA** | 43.29 ± 1.23 | 42.69 ± 1.58 | 43.07 ± 1.54 | 43.07 ± 2.11 | 43.22 ± 1.62 |
| **% PUFA** | 47.04 ± 1.63 | 46.94 ± 2.26 | 47.10 ± 1.72 | 46.72 ± 2.57 | 46.49 ± 1.95 |
| **% MUFA** | 9.67 ± 1.44 | 10.38 ± 1.10 | 9.83 ± 1.63 | 10.22 ± 1.48 | 10.29 ± 1.28 |
| **% n-3** | 4.81 ± 1.20 | 5.79 ± 1.34 | 5.20 ± 1.49 | 5.09 ± 1.85 | 5.32 ± 1.45 |
| **% n-6** | 42.23 ± 1.88 | 41.14 ± 2.00 | 41.90 ± 2.28 | 41.63 ± 2.58 | 41.16 ± 2.24 |
| **n-3/n-6** | 0.11 ± 0.03 | 0.14 ± 0.30 | 0.13 ± 0.04 | 0.12 ± 0.05 | 0.13 ± 0.04 |
| **(EPA+DHA)/AA** | 0.28 ± 0.10 | 0.43 ± 0.12*** | 0.33 ± 0.13 | 0.32 ± 0.14# | 0.34 ± 0.13# |
| **% Arachidonic Acid** | 13.44 ± 2.21 | 10.95 ± 2.08** | 11.80 ± 2.39* | 12.21 ± 1.84* | 12.41 ± 1.73 |
| **% DGLA** | 3.16 ± 0.65 | 3.65 ± 0.47 | 4.10 ± 1.14* | 3.61 ± 0.91 | 3.65 ± 0.63** |
| **% ALA** | 0.27 ± 0.08 | 0.17 ± 0.14 | 0.27 ± 0.16 | 0.17 ± 0.16 | 0.19 ± 0.13 |
| **% Linoleic Acid** | 24.79 ± 2.32 | 25.62 ± 3.83 | 24.89 ± 4.72 | 24.93 ± 3.02 | 24.21 ± 2.56 |
| **% Oleic Acid** | 8.09 ± 1.35 | 8.71 ± 0.99 | 8.20 ± 1.35 | 8.45 ± 1.25 | 8.58 ± 1.23 |
| **% EPA** | 0.71 ± 0.46 | 1.49 ± 0.71** | 1.01 ± 0.65 | 0.98 ± 0.60# | 1.18 ± 0.66** |
| **% DPA** | 0.92 ± 0.17 | 1.02 ± 0.24 | 1.12 ± 0.18 | 0.96 ± 0.27 | 1.03 ± 0.24 |
| **% DHA** | 3.01 ± 0.79 | 3.12 ± 0.97 | 2.80 ± 1.01 | 2.98 ± 1.33 | 2.92 ± 1.05 |

Supplemental Table 2. **Serum fatty acids by medication use in CD**. Comparisons between between all groups were first assessed by the Kruskal-Wallis test and if P <0.05 then pairwise comparisons were performed between groups with the Mann-Whitney U test. Data is presented by mean ± standard deviation. SFA = Saturated Fatty Acids; MUFA = Monounsaturated Fatty Acids; PUFA = Polyunsaturated Fatty Acids; EPA = Eicosapentaenoic acid; DPA = Docosapentaenoic acid; DHA = Docosahexaenoic acid. *P < 0.05, ** P < 0.01, **P < 0.001 vs control. # P<0.05 vs no CD specific therapy. There was no signficance in pairwise comparisons of anti-TNF or combination therapy vs. immunomodulator alone or in anti-TNF alone vs combination therapy. Subjects on corticosteroid alone (n = 1), 5-ASA agents alone (n=6), vedolizumab (n=1), or ustekinumab (n=6) were not analyzed due to small sample size.

|  | **Control**  **(n = 27)** | **Inactive CD**  **(n = 71)** | **Active CD**  **(n = 38)** |
| --- | --- | --- | --- |
| **GM-CSF (pg/mL)** | 3.5 ± 3.2 | 18.1 ± 18.5** | 27.1 ± 32.8** |
| **Lipocalin-2 (ng/mL)** | 287.8 ± 143.6 | 473.0 ± 262.5* | 494. 8 ± 262.2** |
| **CCL11 (pg/mL)** | 140.3 ± 86.8 | 215.9 ± 126.4* | 306.4 ± 210.3** |
| **Resistin (ng/mL)** | 33.0 ± 12.9 | 43.0 ± 21.7 | 49.4 ± 19.9** |
| **GRO (pg/mL)** | 899.5 ± 428.0 | 1177.1 ± 728.0 | 1479.3 ± 1079.3* |
| **VEGF (pg/mL)** | 139.8 ± 106.7 | 287.8 ± 219.6* | 333.7 ± 325.0 |
| **IL-17A (pg/mL)** | 1.8 ± 1.6 | 7.3 ± 10.6 | 14.4 ± 24.1 |
| **IL-7 (pg/mL)** | 2.7 ± 2.9 | 5.9 ± 6.2 | 7.5 ± 7.7 |
| **NGF (pg/mL)** | 1.9 ± 1.5 | 3.0 ± 4.1 | 5.8 ± 8.1 |
| **FGF-2 (pg/mL)** | 60.3 ± 48.3 | 75.4 ± 56.0 | 86.5 ± 45.0 |
| **MDC (pg/mL)** | 971.2 ± 357.8 | 1102.2 ± 438.7 | 1190.3 ± 543.7 |
| **Hepatocyte Growth Factor (pg/mL)** | 460.8 ± 235.7 | 696.3 ± 357.9* | 669.1 ± 546.8 |
| **IP-10 (pg/mL)** | 361.2 ± 124.4 | 307.9 ± 178.9 | 505.9 ± 362.0 |
| **Adipsin (ng/mL)** | 3016.5 ± 610.4 | 2663.4 ± 665.7 | 3060.2 ± 1440.6 |
| **IL-12p40 (pg/mL)** | 25.2 ± 26.0 | 97.8 ± 118.9 | 160.5 ± 219.9 |
| **G-CSF (pg/mL)** | 16.8 ± 19.8 | 37.0 ± 37.5 | 37.8 ± 44.9 |
| **IL-5 (pg/mL)** | 2.4 ± 2.8 | 11.9 ± 18.1 | 19.0 ± 29.7 |
| **IL-8 (pg/mL)** | 8.9 ± 7.9 | 16.4 ± 18.8 | 18.3 ± 23.2 |
| **IL-9 (pg/mL)** | 1.1 ± 0.7 | 3.9 ± 4.1 | 4.2 ± 4.3 |
| **IL-10 (pg/mL)** | 3.2 ± 2.4 | 15.5 ± 20.5 | 15.3 ± 23.7 |
| **IFN-γ (pg/mL)** | 10.2 ± 10.6 | 35.5 ± 41.8 | 34.2 ± 40.1 |
| **Adiponectin (ng/mL)** | 36290.8 ± 34202.6 | 30820.7 ± 44436.2 | 28999.2 ± 27971.8 |
| **MCP-1 (pg/mL)** | 427.4 ± 120.9 | 513.4 ± 222.6 | 504.1 ± 312.3 |
| **MIP-1α (pg/mL)** | 12.0 ± 6.6 | 40.8 ± 43.8 | 78.0 ± 99.6 |
| **MIP-1β** **(pg/mL)** | 20.8 ± 12.7 | 37.8 ± 36.6 | 41.1 ± 44.9 |
| **MCP3 (pg/mL)** | 120.8 ± 92.3 | 238.1 ± 261.1 | 415.2 ± 630.7 |
| **PAI-1 Total (ng/mL)** | 69.2 ± 22.5 | 79.4 ± 26.9 | 73.7 ± 21.9 |
| **TGF-α (pg/mL)** | 10.4 ± 20.1 | 4.8 ± 6.5 | 4.7 ± 4.4 |
| **Fractalkine (pg/mL)** | 160.6 ± 136.3 | 217.3 ± 301.9 | 216.9 ± 271.8 |
| **IL-1α (pg/mL)** | 38.3 ± 49.9 | 104.5 ± 112.7 | 133.3 ± 132.2 |
| **IL-1ra (pg/mL)** | 43.3 ± 51.0 | 93.4 ± 211.3 | 124.7 ± 272.7 |
| **IL-6 (pg/mL)** | 2.3 ± 2.6 | 5.7 ± 6.7 | 8.0 ± 10.5 |
| **IL-13 (pg/mL)** | 34.2 ± 30.1 | 76.8 ± 111.2 | 140.2 ± 205.7 |
| **IL-15 (pg/mL)** | 2.4 ± 1.3 | 7.7 ± 9.3 | 9.2 ± 12.8 |
| **SCD40L (pg/mL)** | 6088.7 ± 4605.2 | 5156.6 ± 3208.6 | 4458.5 ± 2381.3 |
| **IFNA2 (pg/mL)** | 69.3 ± 40.0 | 32.2 ± 32.9 | 31.0 ± 27.0 |
| **TNF-α (pg/mL)** | 7.6 ± 3.1 | 13.9 ± 31.3 | 10.9 ± 7.6 |
| **TNF-β (pg/mL)** | 65.1 ± 64.5 | 118.4 ± 141.5 | 294.0 ± 395.8 |
| **Leptin (pg/mL)** | 19081.1 ± 14513.8 | 16053.8 ± 16354.0 | 24763.4 ± 20999.1 |
| **EGF (pg/mL)** | 76.4 ± 45.5 | 94.9 ± 62.0 | 90.9 ± 60.8 |

Supplemental Table 3. **Serum Cytokines and Adipokines in Crohn’s Disease vs. Control Subjects by Clinical Disease Activity**. Data are presented as mean ± standard deviation. Active vs. Control, Inactive vs. Control, and Active vs. Inactive were compared using a Mann-Whitney U test and then a false discovery rate (q value) was used to correct for multiple comparisons. Clinically active disease was defined by a Harvey Bradshaw Index (HBI) > 4 and clinically inactive disease by HBI ≤ 4.  *q <0.05, **q < 0.01 versus control. No cytokine reached significance in active vs. inactive with the false discovery rate comparison. Two patients were missing cytokine values. Five patients were missing HBI values. Analytes that resulted in >70% of values in serum being below the lower limit of assay were excluded from further analysis (IL-1β, FLT-3L, IL-12p70, IL-2, IL-3, IL-4).

|  | **Control**  **n = 27** | **CD**  **n = 40** | **q-value** |
| --- | --- | --- | --- |
| **GM-CSF (pg/mL)** | 3.54 ± 3.21 | 21.59 ± 21.46 | 0.002 |
| **CCL11 (pg/mL)** | 140.26 ± 86.75 | 280.27 ± 188.12 | 0.002 |
| **IL-17A (pg/mL)** | 1.80 ± 1.56 | 9.04 ± 8.71 | 0.002 |
| **G-CSF (pg/mL)** | 16.76 ± 19.84 | 50.29 ± 44.16 | 0.005 |
| **IL-5 (pg/mL)** | 2.39 ± 2.80 | 17.19 ± 24.48 | 0.011 |
| **IL-7 (pg/mL)** | 2.70 ± 2.86 | 7.52 ± 7.20 | 0.009 |
| **IL-8 (pg/mL)** | 8.88 ± 7.87 | 24.08 ± 25.98 | 0.006 |
| **MCP-1 (pg/mL)** | 427.41 ± 120.86 | 596.48 ± 276.11 | 0.011 |
| **MIP-1α (pg/mL)** | 12.04 ± 6.59 | 47.76 ± 59.30 | 0.011 |
| **MIP-1β (pg/mL)** | 20.79 ± 12.66 | 47.36 ± 40.60 | 0.005 |
| **TNF-α (pg/mL)** | 7.56 ± 3.06 | 13.47 ± 10.85 | 0.008 |
| **VEGF (pg/mL)** | 139.77 ± 106.69 | 312.47 ± 210.70 | 0.005 |
| **Lipocalin-2 (ng/mL)** | 287.80 ± 143.63 | 442.45 ± 242.37 | 0.011 |
| **PAI-1 Total (ng/mL)** | 69.21 ± 22.53 | 85.62 ± 25.05 | 0.009 |
| **FGF-2 (pg/mL)** | 60.33 ± 48.33 | 82.99 ± 45.35 | 0.015 |
| **TGF-α (pg/mL)** | 10.38 ± 20.12 | 4.25 ± 2.56 | 0.008 |
| **IFN-γ (pg/mL)** | 10.17 ± 10.58 | 44.87 ± 44.21 | 0.014 |
| **GRO (pg/mL)** | 899.50 ± 428.03 | 1141.59 ± 527.64 | 0.024 |
| **IL-9 (pg/mL)** | 1.07 ± 0.67 | 4.02 ± 4.73 | 0.027 |
| **IL-10 (pg/mL)** | 3.21 ± 2.43 | 18.38 ± 24.53 | 0.027 |
| **IL-12p40 (pg/mL)** | 25.23 ± 25.96 | 97.34 ± 130.47 | 0.016 |
| **IL-15 (pg/mL)** | 2.36 ± 1.28 | 9.28 ± 11.03 | 0.015 |
| **Resistin (ng/mL)** | 33.03 ± 12.93 | 44.80 ± 20.77 | 0.014 |

Supplemental Table 4. **Significant Serum Cytokines and Adipokines in Crohn’s Disease vs. Control Subjects After Exclusion of Subjects on an Anti-TNF-α Agent**. Data are presented as mean ± standard deviation. The Mann-Whitney U test was used to calculate p value, then false discovery rate (q value) was determined by the Benjamini and Hochberg method. Analytes that resulted in >70% of values in serum being below the lower limit of assay were excluded from further analysis (IL-1β, FLT-3L, IL-12p70, IL-2, IL-3, IL-4). Only cytokines or adipokines with q < 0.05 are shown.
